# Outcomes of Self-Expanding Covered Stents for the Treatment of External ILIAC Artery Obstructive Disease

**DOI:** 10.1007/s00270-023-03370-9

**Published:** 2023-02-24

**Authors:** Francesco Squizzato, Vicente Mosquera-Rey, Amer Zanabili Al-Sibbai, Lino Antonio Camblor Santervas, Edoardo Pasqui, Giancarlo Palasciano, Gianmarco de Donato, Manuel Alonso Pérez, Michele Antonello, Michele Piazza

**Affiliations:** 1 Division of Vascular and Endovascular Surgery, Department of Cardiac, Thoracic, Vascular Sciences and Public Health, University of Padua, Padua, Italy; 2 Angiology and Vascular Surgery Department, Hospital Universitario Central de Asturias (HUCA), Oviedo, Spain; 3 Division of Vascular Surgery, University of Siena, Siena, Italy

**Keywords:** Iliac artery, Stent, Self-expandable metallic stent, Peripheral arterial disease, Multicenter study

## Abstract

**Purpose:**

To describe the early results and mid-term patency rates of external iliac artery (EIA) stenting using self-expanding covered stents.

**Methods:**

We conducted a multicenter retrospective study (2015–2021), including patients receiving primary endovascular treatment of external iliac artery occlusive disease with self-expanding covered stents. All patients were treated with the Viabahn (W.L Gore & Associates, Inc., Flagstaff, AZ–USA) stent. Patency and limb salvage rates were estimated with Kaplan–Meier curves.

**Results:**

Ninety-three patients (mean age, 69 ± 9 years; 81% males) were treated for disabling claudication in 44%, rest pain in 28%, and tissue loss in 28%. TASC C/D lesions were present in 72% and iliac complete occlusion in 30%. Mean lesion length was 6.9 ± 2.4 cm; 30% had moderate/severe EIA calcifications; and the mean iliac tortuosity index was 1.17 ± 0.13. Technical success was 100%. There was one perioperative death (1.4%) and procedural complication rate was 6.5%. At 42 months (mean, 25 months), primary patency was 89.8% (95%CI 83–98); the presence of EIA tortuosity (tortuosity index > 1.25, 87.7 ± 11% vs 89.9 ± 8%; *P* = .6) or severe calcifications (87.6 ± 9% vs 96.0 ± 8%; *P* = .400) had no significant impact. After univariate analysis, the use of a stent with diameter < 8 mm (HR 8.5, 95%CI 3.24–14.22; *P* < .001) was negatively associated with primary patency.

**Conclusions:**

The use of self-expanding covered stents provided excellent early and mid-term results in the treatment of obstructive disease of the EIA, also in cases of high EIA tortuosity and high grade of calcifications. The use of a < 8 mm-diameter stent was associated with a reduced primary patency.

## Introduction

Endovascular treatment represents today a valid option for iliac artery occlusive disease, owing to the low invasiveness, short hospitalization, reduced risk of perioperative complications, and acceptable patency rates [1]. The evolution of materials, with introduction of dedicated guidewires, more flexible low-profile stents, and different stent designs, has allowed to progressively expand the indications also to more complex iliac artery lesions traditionally treated by open surgery, as those classified as TASC C and D, occlusions, long lesions involving both the common and external iliac arteries, or heavily calcified lesions [2–8].

The endovascular treatment of external iliac artery (EIA) may warrant some specific considerations. Compared to common iliac artery (CIA), the EIA is characterized by a smaller diameter, a more tortuous anatomy, and a more frequent extension of the disease into the common femoral artery (CFA) [9, 10]. Also, specific mechanical bending and shortening/elongation forces along with the hip flexion [11] that may exert an adjunctive stress on the materials used for EIA stenting.

For these reasons, the choice of the type of stent for EIA treatment is of paramount importance, as it should guarantee a great conformability to EIA tortuosity and plasticity, adapt to movements without the risk of fracture or infolding, and prevent intimal hyperplasia. However, there is no consensus on the specific type of stent to be used [12]. The aim of this study is to report the early and mid-term results of a multicentric experience in the endovascular treatment of EIA obstructive disease, using the Viabahn^®^ self-expanding PTFE-covered stent (W.L Gore & Associates, Inc., Flagstaff, AZ–USA).

## Methods

### Study Design

We conducted a multicenter retrospective cohort study prospectively collecting data from three tertiary centers in Italy and Spain (January 2015–January 2021). One center was responsible for merging the data, quality check, and requiring audits as needed. Part of the patients (*n* = 21, 22%) in this study cohort had been included in previously published series [10]. Approval for this e study was obtained from each center’s institutional review board.

### Inclusion Criteria

Only patients with iliac obstructive disease of the EIA treated with the Viabahn stent graft were included. Patients with previous endovascular/ open EIA procedures, associated aneurysm, or treated with different types of stent were excluded. Patients receiving stenting of the common iliac artery were also excluded.

### Definitions

Demographics, cardiovascular risk factors, and presenting symptoms were evaluated. Indications to treatment were lifestyle-limiting claudication, rest pain, or ischemic tissue loss [1, 13–15].

**Fig. 1:**
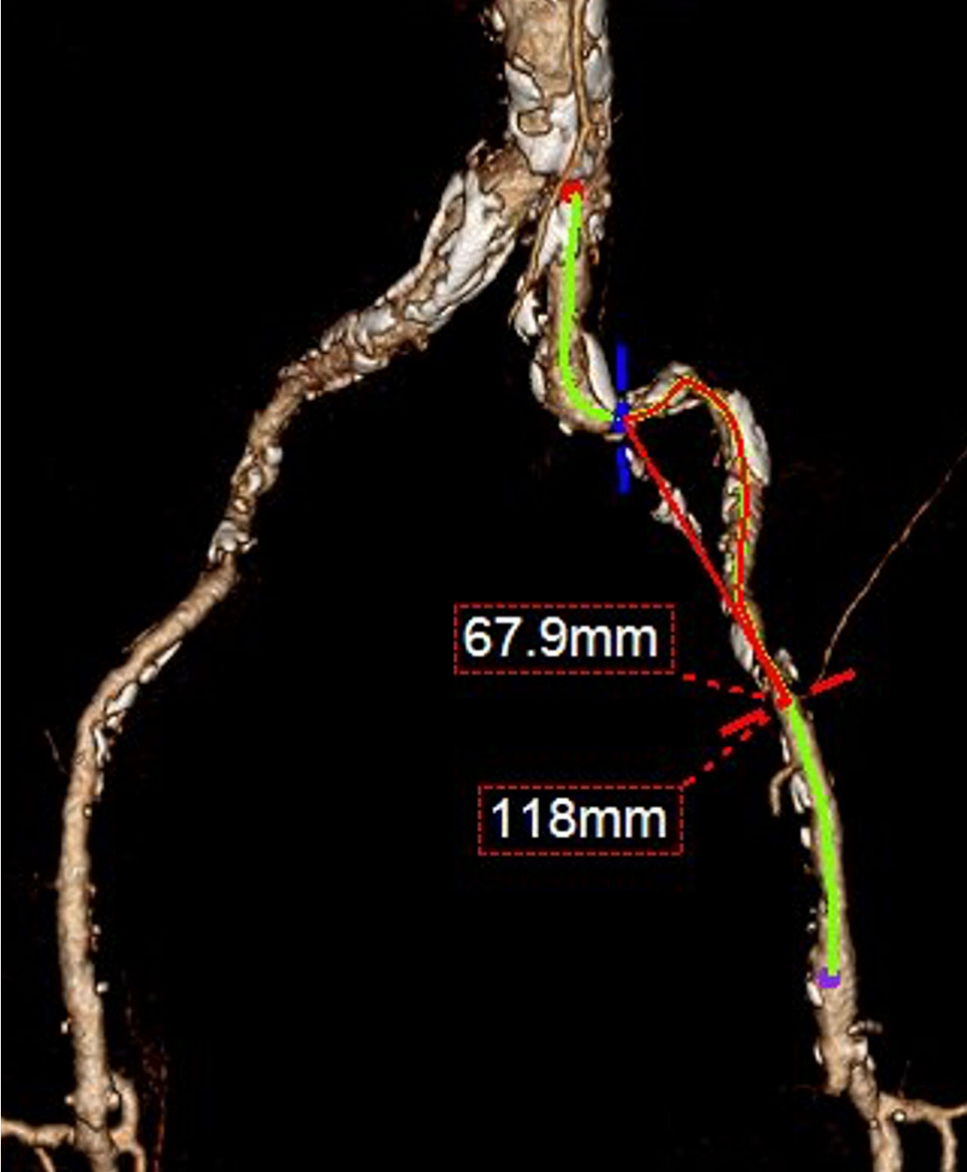
Three-dimensional CTA reconstruction of a left EIA disease. The tortuosity index is measured as the ratio between the centerline and the straight-line distance from the EIA origin to the circumflex arteries

Data on Trans-Atlantic Inter-Society Consensus (TASC) II classification, disease extension grade of calcification, and iliac tortuosity index were obtained from the computed tomography angiography (CTA). Calcifications were categorized as none (< 25% circumference), mild (26–50%), moderate (51–75%), or severe (> 75%). Tortuosity index was measured as the ratio between the centerline and the straight-line distance from the EIA origin to the circumflex arteries [16], measured on 3D CTA reconstructions (Fig. 1).

Technical success was defined on an intention-to-treat basis, as the successful recanalization and stent deployment, with a residual stenosis < 30%. Early (30-days) medical complications included death, myocardial infarction, dysrhythmia, respiratory failure, and acute renal. Procedural complications included arterial access complications needing treatment, bowel ischemia, distal embolization, thrombosis, or wound complications occurring intraoperatively or within 30 days.

Follow-up included clinical examination with ABI and duplex ultrasound at 1, 6, and 12 months and then yearly. Ultrasound criteria for a significant restenosis were a peak systolic velocity (PSV) > 275 cm/s or velocity ratio (PSV at the site of the stenosis/PSV in a normal vessel segment proximal to the stenosis) > 3 [17]. CTA was performed as needed. Primary patency, secondary patency, primary assisted patency, and limb salvage were defined according to the current reporting standards [13].

### Operative Technique

The preferred vascular access was the ipsilateral CFA. The access was percutaneous if the CFA was free from atherosclerotic c plaque (< 30%) on the anterior wall, differently a groin cut-down was performed. The Perclose Proglide (Abbott, Chicago, IL-USA) was preferentially used as percutaneous closure device. When the disease extended into the CFA with a stenosis > 50%, endarterectomy with patch angioplasty was routinely performed, using a great saphenous vein or bovine pericardial patch. In cases of unsuccessful recanalization from the CFA or in most challenging cases, a contralateral CFA or left brachial artery access was adopted for recanalization.

**Fig. 2:**
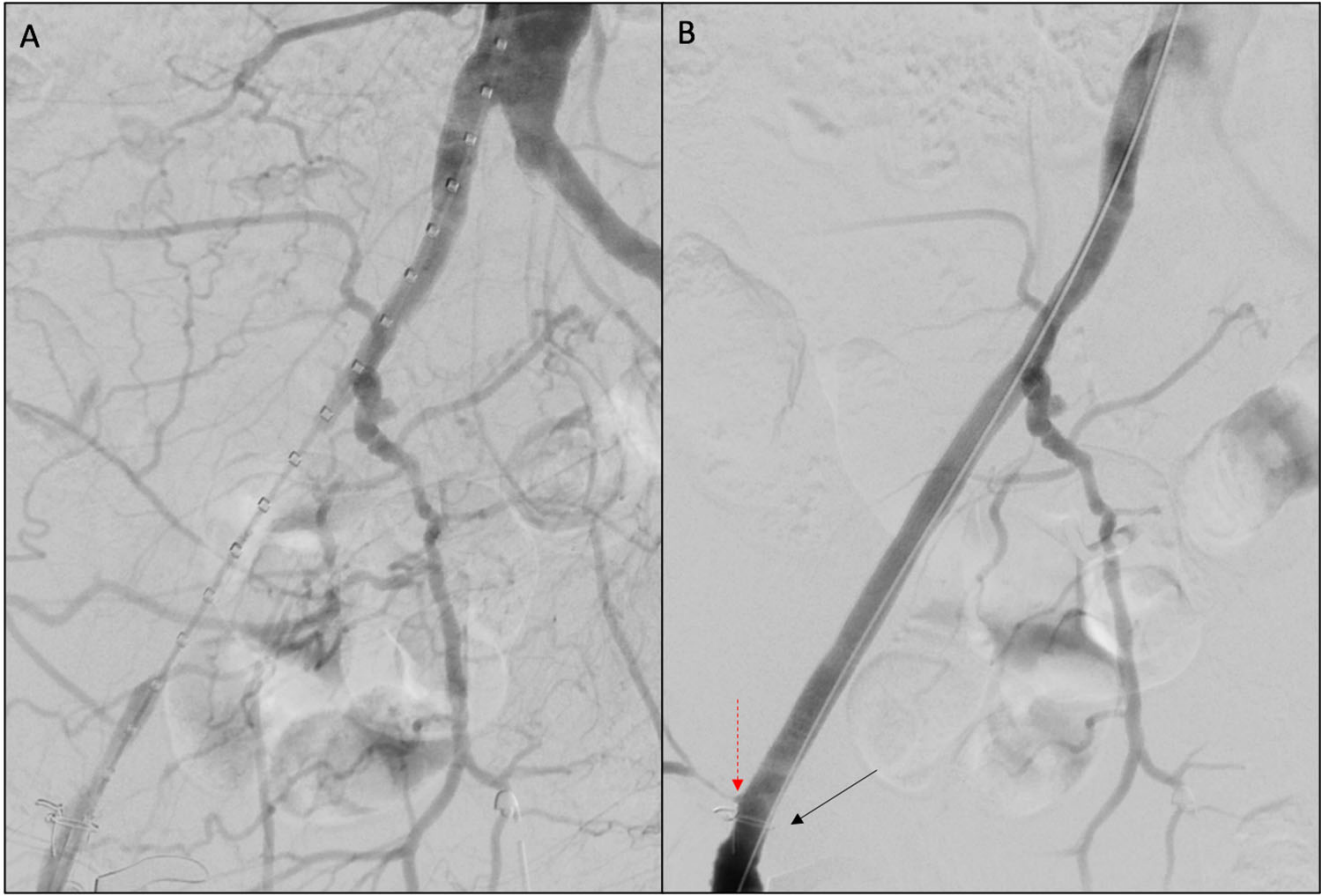
Intraoperative images of a right EIA recanalization and stenting after CFA endarterectomy. **A** Intraoperative angiography showing a right EIA occlusion. **B** Final result after coverage of the entire EIA length with a Viabahn stent. To note the radiopaque marker (black solid arrow) placed during the surgical endarterectomy at the level of the proximal end of the patch plasty, to facilitate the identification of the distal landing zone for the stent. Alternatively, the circumflex arteries (red dashed arrow) may be used as a distal reference for the deployment of the stent

After lesion crossing, a stiff guidewire was advanced from the ipsilateral CFA. In case of recanalization from the brachial or contralateral femoral access, the guidewire was usually snared from the ipsilateral CFA and then exchanged for a stiff guidewire, and the stent was deployed from the ipsilateral CFA as usual. In a minority of percutaneous cases (*n* = 6) with EIA disease extending till the level of the circumflex arteries, the stent was deployed from the contralateral CFA access after establishment of a femoral–femoral through-and-through. Predilatation with small diameter balloons (4–6 mm) was usually performed in chronic total occlusions. Then the Viabahn stent was deployed covering the entire lesion length (Fig. 2). The stents were sized on the diameter of the native treated artery (with 1 mm oversize) and deployed landing in a tract of healthy artery. If feasible, a single stent was used to cover the entire lesion. If multiple stents were required, these were overlapped for at least 1 cm. Post-dilatation was performed using non-compliant balloon. In case of associated CFA endarterectomy, the distal edge of the stent was deployed till the level of the iliac circumflex and inferior epigastric arteries, to cover the intimal flap of the endarterectomized artery.

In case of multilevel disease involving both the iliac and femoropopliteal districts, only the proximal (iliac) segment was treated in patients presenting with intermittent claudication, rest pain, or small superficial foot lesions; the treatment of both the iliac and femoropopliteal obstructions was performed in cases with ischemic tissue loss.

Intravenous heparin was administered intraoperatively to elevate activated clotting time > 250 s. After the intervention, dual antiplatelet therapy (aspirin 100 mg and clopidogrel 75 mg daily) was prescribed for at least 1 month [1].

### Statistical Analysis

Continuous data are presented as mean ± standard deviation or median (range), categorical data as number and percentage. Kaplan–Meier survival curves for primary patency, secondary patency, primary-assisted patency, and limb salvage were estimated. Univariate Cox proportional hazards were used to identify clinical, anatomical, and procedural factors associated with primary patency. A penalized likelihood method based on Firth’s regression [18] was adopted to account for the small number of events. A multivariate analysis was not performed due to the risk of overfitting. All analyses were carried out with the R 4.0 software (R Foundation for Statistical Computing, Vienna, Austria), and a *P*-value < 0.05 (two-tailed) was considered statistically significant.

## Results

Ninety-three patients were treated with deployment of the Viabahn stent in the EIA. Mean age was 69 ± 9 years and 81% were male (Table 1). Symptoms at presentation were disabling claudication in 44%, rest pain in 28%, and tissue loss in 28% (Table 2). Most patients were categorized as TASC II C (bilateral EIA stenosis 3–10 cm long not extending into the CFA, unilateral EIA stenosis extending into the CFA, unilateral EIA occlusion involving the origins of the internal iliac and/or CFA, heavily calcified unilateral EIA occlusion with or without involvement of origins of internal iliac and/or CFA) or D (bilateral occlusion of the EIA) (72%) and iliac occlusion was present in 28 (30%) limbs. Mean lesion length was 6.9 ± 2.4 cm and 30% had moderate/severe calcifications. CFA stenosis > 50% was present in 52% of limbs and femoropopliteal occlusive disease in 61%.

**Table 1:** Demographics and baseline risk factors of the 93 patients treated for isolated external iliac obstructive disease

Variable	N (%) / mean ± SD
*Demographics*	
Age, years	
Mean ± SD	69.5 ± 9.1
Median (range)	68 (51–90)
Male sex	75 (80.6)
*Risk factors*	
Hypertension	77 (82.8)
Diabetes	30 (32.3)
Dyslipidemia	62 (66.7)
Smoking	
Active smoker	31 (33.3)
Former smoker	33 (35.5)
Coronary artery disease	26 (28.0)
Prior CABG	2 (2.2)
Prior PTCA	10 (10.8)
CHF	10 (10.8)
Atrial fibrillation	20 (21.5)
Cerebrovascular disease	4 (4.3)
Renal insufficiency	11 (11.8)
COPD	19 (20.4)
ASA score	2.7 ± 0.5

*CABG* coronary artery bypass graft; *PTCA* percutaneous transluminal coronary angioplasty; *CHF* congestive heart failure; *COPD* chronic obstructive pulmonary disease; *ASA* American Society of Anesthesiologists

**Table 2:** Clinical and anatomical data of the 93 patients treated for external iliac obstructive disease

Variable	N (%) / mean ± SD
*Clinical data*	
Rutherford category	
3	41 (44.1)
4	26 (28.0)
5	23 (24.7)
6	3 (3.2)
*Anatomical data*	
TASC II category	
A	8 (8.6)
B	18 (19.4)
C	54 (58.1)
D	13 (14.0)
Iliac occlusion	28 (30.1)
Lesion length, cm	6.9 ± 2.4
EIA grade of calcifications	
< 25%	49 (52.7)
26–50%	16 (17.2)
51–75%	17 (18.3)
> 75%	11 (11.8)
EIA tortuosity index	1.17 ± 0.13
CFA diameter, mm	7.9 ± 1.2
CFA grade of stenosis	
Minimal (< 50%)	39 (41.9)
Moderate/high (50–74%)	20 (21.5)
High (75–99%)	18 (19.4)
Occlusion	10 (10.8)
Femoropopliteal occlusive disease	57 (61.3)

*CIA*, common iliac artery*; EIA*, external iliac artery*; CFA,* common femoral artery

^a^Statistically significant

Iliac recanalization was performed via retrograde femoral approach in 69% of cases and a contralateral approach in 24%; antegrade recanalization from a brachial access was required in 7.5%. The access was percutaneous in 39%. Mean length of coverage was 9.5 ± 0.2 cm, using a mean number of 1.1 ± 0.3 stents. Stent’s diameter was < 8 mm in 26 patients (28%). Forty-eight (52%) patients required associated CFA endarterectomy and 22 (25%) the concomitant treatment of femoropopliteal disease (femoropopliteal bypass in 17 and PTA in 6). No patients received tibial arteries revascularization.

Thirty-day results are reported in Table 3. Technical success was 100%. There was one perioperative death in a patient who had a postoperative myocardial infarction. Procedural complications included 2 (2.1%) access site pseudoaneurysm requiring reintervention and 4 (4.3%) wound complications. There were no cases of iliac artery rupture, bowel ischemia, or distal embolization.

**Table 3:** Periprocedural data and early outcomes of the 93 patients treated for external iliac obstructive disease, stratified by need for concomitant CFA endarterectomy

Variable	Total (N (%) / mean ± SD)	Isolated EIA stenting	EIA stenting + CFA endarterectomy	*P*
Procedural data	*N* = 93	*n* = 45	*n* = 48	
Vascular access				
Femoral ipsilateral	64 (68.8)	26 (57.8)	38 (79.2)	.042^a^
Femoral contralateral	22 (23.6)	14 (31.1)	8 (16.7)	.143
Brachial	7 (7.5)	5 (11.1)	2 (4.2)	.257
Percutaneous	36 (38.7)	36 (80.0)	0 (0)	< .001^a^
Mean number of stents	1.1 ± 0.3	1.1 ± 0.4	1.2 ± 0.3	.737
Length of coverage, cm	9.5 ± 3.2	9.4 ± 3.3	9.6 ± 3.1	.709
Mean stent diameter, mm	7.8 ± 0.8	7.8 ± 0.8	7.9 ± 0.9	.697
6 mm	3 (3.2)	1 (2.2)	2 (4.4)	.631
7 mm	23 (24.7)	11 (24.2)	12 (25.0)	
8 mm	58 (62.4)	28 (62.2)	30 (62.5)	
9 mm	2 (2.2)	2 (4.4)	0 (0)	
10 mm	7 (7.5)	3 (6.7)	4 (8.3)	
Associated procedures	55 (59.1)			
CFA endarterectomy	48 (51.6)	0 (0)	48 (100)	-
Femoropopliteal bypass	17 (18.3)	5 (11.1)	12 (25.0)	.109
SFA PTA/stent	6 (6.5)	3 (6.7)	3 (6.3)	1.00
Medical outcomes				
Major cardiac	1 (1.4)	0 (0)	1 (2.1)	1.00
Respiratory failure	0 (0)	0 (0)	0 (0)	1.00
Acute kidney injury	2 (2.1)	2 (4.4)	1 (2.1)	.234
Dialysis	0 (0)	0 (0)	0 (0)	1.00
Death	1 (1.4)	0 (0)	1 (2.1)	1.00
Procedural outcomes				
Cumulative procedural complications rate	6 (6.5)	1 (2.2)	5 (10.4)	.204
Iliac rupture	0 (0)	0 (0)	0 (0)	1.00
Distal embolization	0 (0)	0 (0)	0 (0)	1.00
Bowel ischemia	0 (0)	0 (0)	0 (0)	1.00
Access site pseudoaneurysm	2 (2.1)	1 (2.2)	1 (2.1)	1.00
Wound infection/dehiscence	4 (4.3)	0 (0)	4 (8.3)	.117

*CFA,* common femoral artery; SFA, superficial femoral artery

^a^Statistically significant

**Fig. 3:**
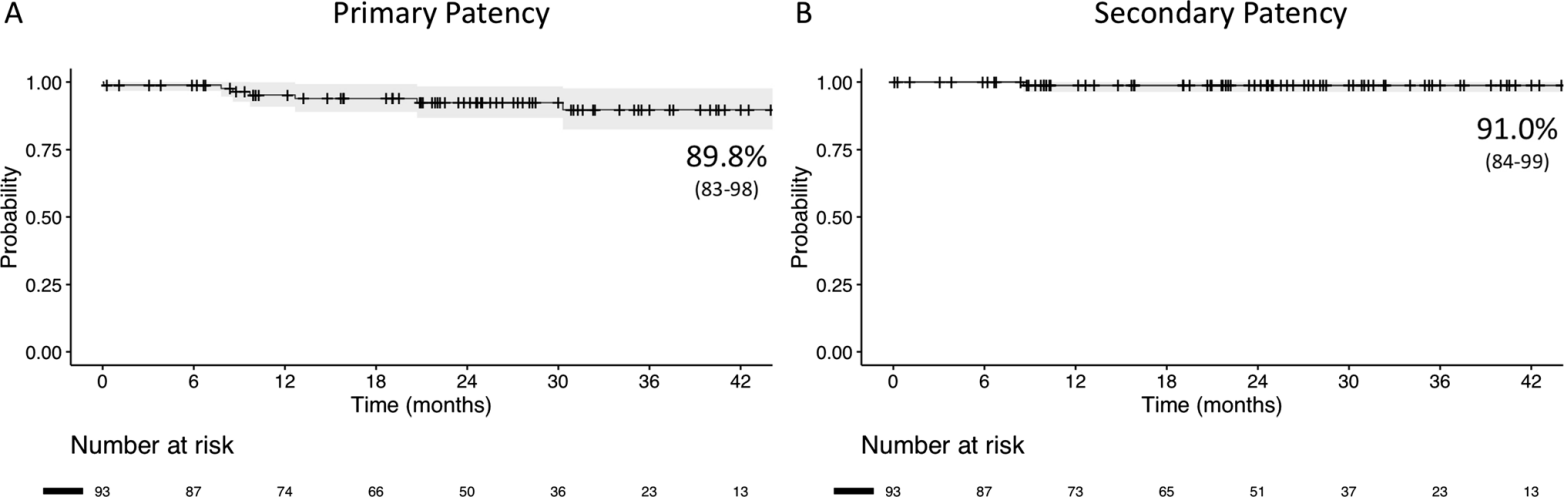
**A** Kaplan-Meier estimate of primary patency after stenting of the EIA using self-expanding covered stents. Standard Error <10%. **B** Kaplan-Meier estimate of secondary patency after stenting of the EIA using self-expanding covered stents. Standard Error <10%

Median follow-up was 25 months. Overall survival was 70.1% (95%CI 59–83) at 42 months. During follow-up, there were 7 stent occlusion or restenosis; 3 were treated with re-stenting using covered stents, 1 with fibrinolysis and re-stenting, 1 with thrombectomy, 2 with re-do CFA endarterectomy and EIA re-PTA and stenting. Estimated primary patency at 42 months was 89.8% (95%CI 83–98) and the primary-assisted patency was 91% (95%CI 84–98). Primary patency was similar also in cases with high EIA tortuosity (tortuosity index > 1.25, 87.7 ± 11%) compared with low tortuosity (tortuosity index ≤ 1.25, 89.9 ± 8%; *P* = 0.600) and in cases with highly calcified lesions compared with less calcified iliacs (87.6 ± 9% vs 96.0 ± 8%; *P* = 0.400). Secondary patency was 91.0% (95%CI 84–99) and limb salvage was 94.6% (95%CI 60–100) (Fig. 3).

Comparing patients receiving isolated EIA stenting vs those receiving EIA stenting + CFA endarterectomy, technical success (100% vs 100%; *P* = 1.00), medical complication rate (4.4% vs 6.3%; *P* = 1.00), and procedural complication rate (2.2% vs 10.4%; *P* = 0.117) were not significantly different. Primary patency was 87.7% (95%CI 76–100) vs 92.6% (95%CI 85–100; *P* = 0.780), secondary patency was 97.6% (95%CI 93–100) vs 100% (*P* = 0.320), and limb salvage was 91.7% (95%CI 80–100) vs 97.8% (93–100; *P* = 0.400).

After univariate analysis, the use of a stent diameter < 8 mm (HR 8.5, 95%CI 3.24–14.22; *P* < 0.001) was negatively associated with patency (Table 4). Overall patients baseline clinical, anatomical, and procedural characteristics were similar between patients with stent diameter ≥ 8 mm vs < 8 mm (Table 5). The presence of iliac occlusion rather than a stenosis (HR 3.01, 95%CI 0.73–13.50), calcifications involving > 50% arterial circumference (HR 6.29, 95%CI 0.94–25.80; *P* = 0.068), TASC classification (TASC D, HR 0.32, 95%CI 0.02–2.64; *P* = 0.356), and associated outflow disease (HR 1.17, 95%CI 0.28–2.57; *P* = 0.366) were not significantly associated with primary patency.

**Table 4:** Univariate Cox proportional hazards for primary patency

	HR (95%CI)	*P*
*Clinical factors*		
Female sex	0.69 (0.08–5.72)	.727
Age, years	0.92 (0.83–1.13)	.092
Rutherford 5/6	1.19 (0.21–4.99)	.815
Smoke	1.64 (0.37–6.78)	.489
*Anatomical factors*		
Iliac occlusion	3.01 (0.73–13.50)	.122
TASC D	0.32 (0.02–2.64)	.356
Lesion length, cm	1.05 (0.78–1.41)	.761
Iliac calcification > 50%	6.29 (0.94–25.8)	.068
Femoropopliteal occlusive disease	1.17 (0.28–6.57)	.829
EIA tortuosity index	0.35 (0.02–2.57)	.366
*Procedural factors*		
Stent diameter mm	0.41 (0.15–1.17)	.098
Stent diameter < 8 mm	8.51 (3.24–14.22)	< .001^a^
Length of coverage, mm	1.05 (0.84–1.29)	.635
Number of stents	0.32 (0.02–2.70)	.368
Associated CFA endarterectomy	0.83 (0.18–3.44)	.802
Any associated procedure	1.33 (0.32–7.41)	.703

*CFA,* common femoral artery; *SFA*, superficial femoral artery

^a^Statistically significant

**Table 5:** Comparative analysis between patients with stent diameter ≥ 8 mm vs < 8 mm

	Stent diameter ≥ 8 mm *n* = 67	Stent diameter < 8 mm *n* = 26	P
*Clinical factors*			
Female sex	10 (14.9)	8 (30.8)	.083
Age, years	69 ± 9	69 ± 10	.988
Rutherford 5/6	16 (23.8)	10 (38.5)	.199
Smoke	44 (65.7)	20 (76.9)	.330
*Anatomical factors*			
Iliac occlusion	24 (35.8)	4 (15.4)	.054
TASC D	11 (16.4)	2 (7.7)	.340
Lesion length, cm	7.1 ± 2.4	6.7 ± 2.1	.456
Iliac calcification > 50%	23 (34.3)	8 (30.8)	.810
Femoropopliteal occlusive disease	38 (56.7)	19 (73.1)	.146
EIA tortuosity index	1.17 ± 0.11	1.16 ± 0.14	.645
*Procedural factors*			
Stent diameter mm	8.2 ± 0.6	6.8 ± 0.3	< .001^a^
Length of coverage, mm	9.7 ± 3.3	9.1 ± 2.7	.407
Number of stents	1.1 ± 0.4	1.1 ± 0.3	.677
Associated CFA endarterectomy	34 (50.7)	14 (53.8)	.948
Any associated procedure	43 (64.2)	16 (61.5)	.812

^a^Statistically significant

## Discussion

Specific patency rates after endovascular treatment of obstructive atherosclerotic disease of the EIA are not well defined. Timaran et al. [9] in 2001 reported worsened outcomes after stenting of the EIA compared to the CIA, with a 23% vs 72% primary patency at 5 years. However, only uncovered old-generation stents were used, and both balloon and self-expanding stents were merged together. Another prior experience [10] reported that lesions involving the EIA carry a twofold chance of stent failure in female patients (*P* = 0.048). Also, worsened patency rates have been reported for lesions involving both the CIA and EIA, and this may be more related to complications occurring at the level of the EIA rather than the CIA [3].

In this context, there is no consensus regarding the preferred type of stent to be used for EIA [12]. The use of covered stents, bare-metal stents, balloon-expandable, and self-expandable stents is reported in the literature [2, 3, 6, 9, 19–21], but a direct comparison is not available specifically for this district.

The authors’ preference is to use the Viabahn self-expanding covered stent for this anatomical segment. The high flexibility allows to adapt to the EIA tortuous anatomy and to conform to the bending, elongation, and torsion that occur with hip movements. The covered design minimizes the chance of in-stent restenosis and allows for an aggressive post-dilatation avoiding the risk of rupture [4–6]. In this study, we achieved a satisfactory 90% primary patency and 91% secondary patency after 42 months from treatment. These results were obtained in a cohort of patients with a > 70% prevalence of TASC II C/D lesions and with an iliac complete occlusion in 30% of the treated limbs.

To optimize outcomes, we usually follow a few technical tips. To facilitate stent advancement through heavily calcified or occluded vessels, a predilatation with a 4–6-mm balloon is usually needed; an alternative is to place a long adequate-size sheath after recanalization, to avoid any eventual stent-graft dislocation during its advancement. For the EIA, additional attention is paid to prevent the chance of kinking at the proximal or distal edge, where infolding of the stent’s end may occur as a result of arterial tortuosity or bending forces. Therefore, we usually aim to land in a straight and relatively fixed portion of the EIA, covering its entire length.

The CFA represents the direct EIA outflow, and an appropriate treatment of CFA stenosis is mandatory to maintain stent patency over time. A CFA endarterectomy was associated with EIA stenting in approximately 50% of limbs in our cohort. In these cases, the stent is deployed till the level of the endarterectomized artery in order to stabilize the residual intimal flap of the endarterectomy; a radiopaque marker may be placed during the open repair at the level of the proximal end of the patch plasty to facilitate the identification of the distal landing zone for the Viabahn. Differently, the indications to revascularization of concomitant disease of the superficial femoral artery depend on the patient’s clinical presentation, and the presence of femoropopliteal occlusive disease does not seem to impact on EIA stent patency (*P* = 0.829).

**Fig. 4:**
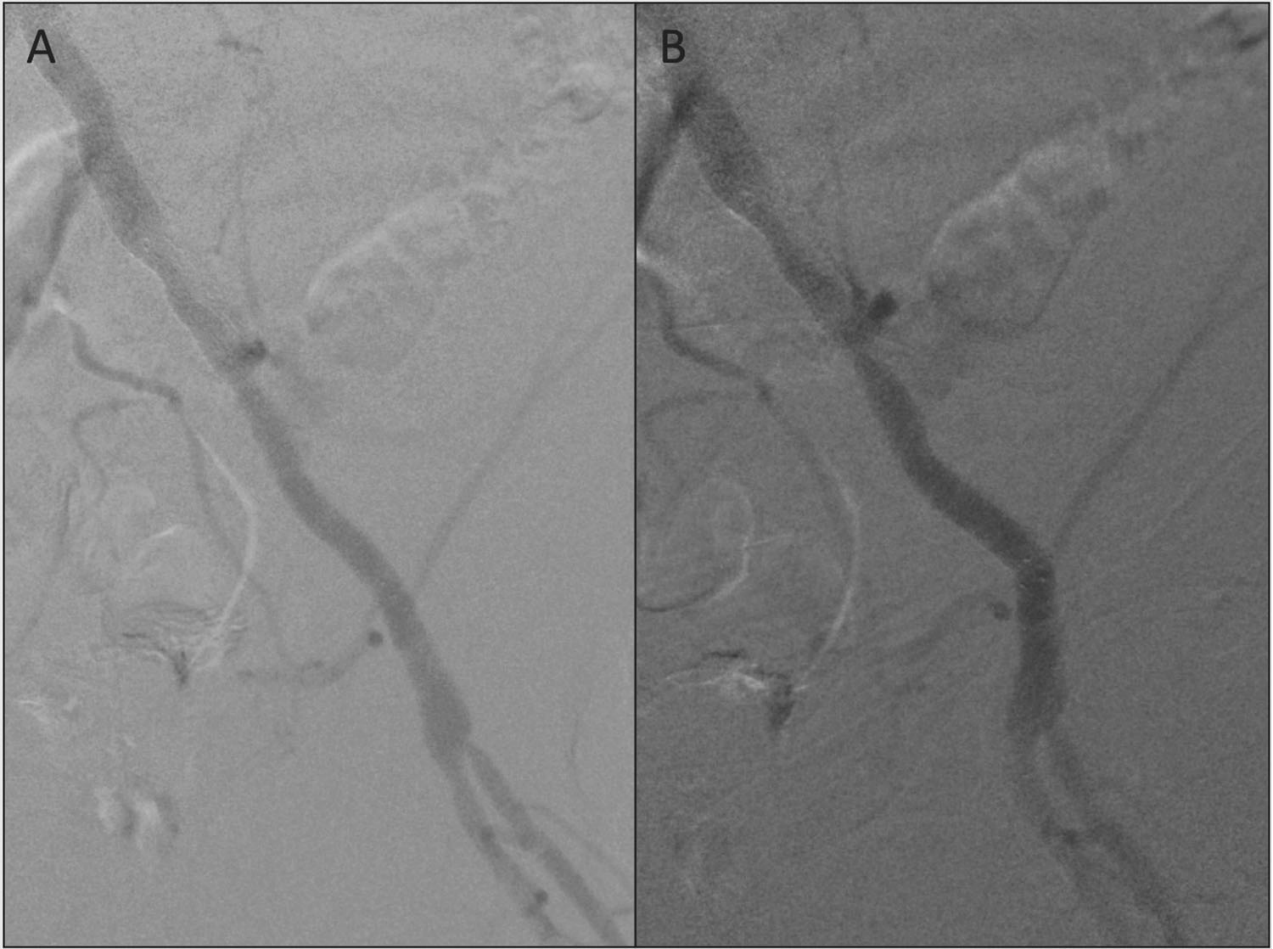
**A** Completion angiogram after successful EIA stenting. **B** Completion angiogram performed during hip flexion, to identify the bending point of the EIA

It is interesting to note that EIA patency was not significantly affected by arterial tortuosity (*P* = 0.366). In cases with high tortuosity, it may be useful to perform the final completion angiogram without the stiff guidewire in place. In addition, when the iliac stent lands in a zone of potential flexion, it may be useful to perform the angiogram after hip flexion (Fig. 4).

According to our results, the presence of small arteries still represents an unfavorable scenario, since the use of a stent diameter < 8 mm was associated with an increased risk of reduced patency. This result is in line with previous data from both monocentric and multicenter studies using covered or uncovered stents [5, 10]. In the EIA the use of a covered stent is protective from iliac rupture, and an 8-mm stent may be safely deployed also in case of native arterial diameter of 6–7 mm. If a smaller stent has to be used for technical or availability reasons, a prolonged dual antiplatelet therapy may be considered.

Excessive arterial calcifications may carry additional procedural difficulties and hinder the mid-term and long-term results. Although our analysis may have been limited, the presence of heavily calcified EIA was not associated with patency. If a residual stenosis persists also after self-expanding stent deployment and standard post-dilatation, in our opinion the use of a more rigid [19] balloon-expandable stent does not represent the best option for the EIA, basing on the concept that conformability to the native anatomy should be respected to optimize the outcomes. We prefer instead to perform a “protected” (after stent deployment) aggressive post-dilatation using an ultra-non-compliant balloon or high-pressure balloon, with the aim to disrupt the plaque.

Our study has some limitations that are worth mentioning. This was a non-randomized retrospective study, and the limited number of events may have limited the statistical analysis. A comparative analysis with other stent designs should be made to better guide the clinical decision; however, it has also to be considered that in our centers, the Viabahn represents the first choice for the treatment of the EIA obstructive disease, and the use of other types of bare-metal or covered stents is very restricted, preventing from a fair comparison.

## Conclusion

The use of self-expanding covered stents provided excellent early and mid-term results in the treatment of obstructive disease of the EIA. Satisfactory results were obtained also in cases of high EIA tortuosity and high grade of calcifications. The use of a < 8 mm-diameter stent was associated with a reduced primary patency. Further studies are needed to compare the results with other types of stent that may be used in the EIA.
